# Determinants of osteoarticular involvement in brucellosis: a multicenter retrospective case–control study

**DOI:** 10.3389/fcimb.2026.1921607

**Published:** 2026-10-14

**Authors:** Yakup Gezer, Muhammet Rıdvan Tayşi, Arzu Tarakçı, Esra Sağlam Kandemir, Tuğba Demircioğlu, Hanife Uzar

**Affiliations:** 1 Infectious Diseases and Clinical Microbiology Clinic, University of Health Sciences, Konya City Hospital, Konya, Türkiye; 2 Department of Infectious Diseases and Clinical Microbiology, University of Health Sciences, Ankara Etlik City Hospital, Ankara, Türkiye; 3 Infectious Diseases and Clinical Microbiology Clinic, Çukurova State Hospital, Adana, Türkiye; 4 Infectious Diseases and Clinical Microbiology Clinic, Tekirdag Dr. İsmail Fehmi Cumalioğlu City Hospital, Tekirdağ, Türkiye; 5 Republic of Türkiye Ministry of Health Gaziantep Provincial Health Administration, Gaziantep, Türkiye

**Keywords:** arthritis, brucellosis, erythrocyte sedimentation rate, osteoarticular involvement, sacroiliitis, spondylodiscitis, symptom duration

## Abstract

**Objective:**

Osteoarticular involvement is the most common focal complication of brucellosis and usually requires prolonged treatment. However, data regarding the factors associated with osteoarticular involvement remain limited. This multicenter retrospective study aimed to identify the clinical and laboratory factors associated with osteoarticular involvement in patients with brucellosis.

**Methods:**

This multicenter retrospective case-control study included adult patients diagnosed with brucellosis at three centers between October 2020 and September 2025. Patients with osteoarticular involvement were defined as cases, whereas patients without osteoarticular involvement served as controls. Demographic, clinical, laboratory, and radiological data were retrospectively collected. Factors associated with osteoarticular involvement were investigated using univariate and multivariable logistic regression analyses.

**Results:**

A total of 804 patients with brucellosis were included in the study. The median age was 45 years, and 516 (64.2%) were male. Osteoarticular involvement was identified in 217 (27.0%) patients. In the multivariable logistic regression analysis, symptom duration (OR: 1.102; 95% CI: 1.068–1.136; p<0.001) and erythrocyte sedimentation rate (ESR) (OR: 1.025; 95% CI: 1.016–1.034; p<0.001) were independently associated with osteoarticular involvement. Receiver operating characteristic (ROC) analysis identified optimal cut-off values of 3.5 weeks for symptom duration and 22.5 mm/hour for ESR in predicting osteoarticular involvement.

**Conclusion:**

Symptom duration and ESR were independently associated with osteoarticular involvement in patients with brucellosis. These readily available clinical and laboratory parameters may facilitate the early identification of patients at high risk for osteoarticular involvement and assist in planning appropriate diagnostic evaluations.

## 1 Introduction

Brucellosis is a systemic infectious disease caused by facultative intracellular Gram-negative bacteria of the *Brucella* species and is recognized as one of the most prevalent zoonotic infections worldwide (Pappas et al., 2006). The disease is endemic in the Mediterranean basin, the Middle East, South America, and certain regions of Asia, posing a substantial burden on both public health and the livestock industry (Pappas et al., 2006; Qureshi et al., 2023). Transmission typically occurs through direct contact with infected animal tissues or body fluids, consumption of unpasteurized milk and dairy products, or inhalation of infectious aerosols (Franco et al., 2007; Narimisa et al., 2024).

The diagnosis of brucellosis is based on the combination of a compatible epidemiological exposure history and clinical manifestations, together with the detection of *Brucella* species by serological testing or culture methods (Yagupsky et al., 2019). The disease follows a systemic course characterized by nonspecific symptoms such as fever, malaise, myalgia, arthralgia, fatigue, and sweating, and may involve reticuloendothelial organs, including the liver, spleen, lymph nodes, and bone marrow (National Center for Emerging and Zoonotic Infectious Diseases (U.S.), 2017). The ability of *Brucella* species to survive intracellularly and disseminate hematogenously facilitates the spread of infection to various organ systems and predisposes affected individuals to the development of diverse focal complications (Von Bargen et al., 2012).

Musculoskeletal involvement is the most common focal complication of brucellosis and represents one of the principal determinants of the disease course and prognosis. Osteoarticular brucellosis is characterized by a spectrum of clinical manifestations, including peripheral arthritis, sacroiliitis, and spondylodiscitis (Buzgan et al., 2010; Pu et al., 2024; Pan et al., 2025). Previous studies have reported that the frequency of osteoarticular involvement ranges from 21.4% to 59.2% across different patient series (Bosilkovski et al., 2004; Buzgan et al., 2010; Arslan et al., 2024; Shi et al., 2024; Dalmanoğlu et al., 2026; Gezer and Tayşi, 2026). Vertebral involvement most commonly affects the lumbar region and is particularly prevalent among adults (Ulu-Kilic et al., 2014). Hematogenous dissemination and intracellular persistence facilitate infection of highly vascularized bone and joint tissues, particularly the vertebral endplates, intervertebral discs, and synovial structures (Von Bargen et al., 2012).

Osteoarticular brucellosis is an important clinical problem because of its prolonged treatment requirements and the substantial morbidity. Patients with osteoarticular involvement require a longer duration of treatment than those with uncomplicated disease, with most patients receiving combination antibiotic therapy for at least three months. The prolonged treatment required for osteoarticular brucellosis, together with chronic pain, functional impairment, the need for surgical intervention in selected cases, and rare neurological complications, may adversely affect patients’ quality of life (Ulu-Kilic et al., 2014; Pu et al., 2024; Guenifi et al., 2025). In addition, because the clinical manifestations of brucellosis are nonspecific, diagnostic delays may occur even in endemic regions, and such delays have been associated with the development of complications. Therefore, early diagnosis and appropriate treatment are critical for reducing the risk of chronicity and relapse (Pu et al., 2024; Dadar et al., 2025).

Despite the high prevalence of osteoarticular involvement and its substantial clinical consequences, data regarding the factors associated with osteoarticular involvement in patients with brucellosis remain limited. Most previous studies have evaluated focal complications as a composite outcome rather than specifically examining osteoarticular involvement. Early identification of the factors associated with osteoarticular involvement is important for the timely implementation of appropriate imaging modalities, optimization of treatment duration, and provision of close clinical follow-up. Therefore, this multicenter retrospective case-control study aimed to investigate the clinical and laboratory factors associated with osteoarticular involvement in patients with brucellosis.

## 2 Materials and methods

### 2.1 Study design

This multicenter retrospective case-control study included patients aged ≥18 years who were diagnosed with brucellosis between October 2020 and September 2025 at three healthcare centers in Türkiye (two tertiary care hospitals and one secondary care hospital). Pregnant women, individuals younger than 18 years of age, patients with incomplete medical records, and those with a previous history of brucellosis were excluded from the study. Among patients with brucellosis, those with osteoarticular involvement were classified as cases, whereas those without osteoarticular involvement were classified as controls.

Demographic, clinical, and laboratory data were retrospectively obtained from the electronic medical records available in the hospital information management systems. The collected variables included age, sex, place of residence (rural/urban), history of exposure associated with a risk of brucellosis, presence of comorbidities, clinical signs and symptoms at presentation, and symptom duration. Comorbid conditions included hypertension, diabetes mellitus, coronary artery disease, malignancy, chronic kidney disease, and chronic lung disease; the presence of at least one of these conditions was defined as the presence of comorbidity. Laboratory evaluations included complete blood count, routine biochemical tests, C-reactive protein (CRP), erythrocyte sedimentation rate (ESR), the Brucella serum agglutination test (SAT), and culture studies. Radiological evaluations were performed using ultrasonography (US), computed tomography (CT), and magnetic resonance imaging (MRI). Imaging studies were requested by the attending physicians according to clinical indications, and radiological assessments were based on the available radiology reports.

### 2.2 Definitions

Brucellosis was defined by the presence of at least one of the following standard diagnostic criteria: (1) isolation of *Brucella* spp. from blood or other sterile body fluid/tissue specimens, or (2) a SAT titer of ≥1:160 in the presence of clinical findings compatible with brucellosis or at least a fourfold increase in antibody titer between serum samples obtained 2–3 weeks apart (Buzgan et al., 2010).

Symptom duration was defined as the interval between symptom onset and the diagnosis of brucellosis. Patients with a symptom duration of <8 weeks were classified as having acute brucellosis, whereas those with a symptom duration of ≥8 weeks were classified as having subacute/chronic brucellosis (Ulu-Kilic et al., 2014).

Focal involvement was defined as involvement of a specific organ in patients with confirmed brucellosis, supported by compatible clinical findings and laboratory and/or radiological confirmation. Osteoarticular involvement was defined as the presence of clinical signs of inflammation (pain, swelling, increased local temperature, erythema, and/or functional limitation) affecting the peripheral joints, sacroiliac joints, or vertebral region in patients with brucellosis, with radiological confirmation of involvement. Osteoarticular involvement was categorized as peripheral arthritis, sacroiliitis, or spondylodiscitis. Peripheral arthritis was defined as involvement of any peripheral joint characterized by clinical signs of inflammation and supported by compatible imaging findings. Sacroiliitis was defined as the presence of pain and tenderness over the sacroiliac joint region together with inflammatory changes demonstrated on magnetic resonance imaging. Spondylodiscitis was defined as magnetic resonance imaging evidence of involvement of at least one vertebral body and/or the adjacent intervertebral disc space secondary to Brucella infection. Hepatic involvement was defined as an increase in aspartate aminotransferase (AST) and/or alanine aminotransferase (ALT) levels to at least five times the upper limit of normal and/or a total bilirubin level >2.5 mg/dL that could not be explained by another etiology. Hematological abnormalities were defined as the presence of anemia, leukopenia, leukocytosis, or thrombocytopenia considered to be associated with brucellosis. Hemoglobin levels <12 g/dL in women and <13 g/dL in men were defined as anemia; a leukocyte count >10, 000/mm^3^ as leukocytosis; a leukocyte count <4, 000/mm^3^ as leukopenia; and a platelet count <150, 000/mm^3^ as thrombocytopenia. Genitourinary involvement was defined as epididymo-orchitis in patients presenting with clinical findings such as scrotal pain, swelling, or hyperemia, together with inflammatory changes involving the epididymis and/or testis detected on scrotal ultrasonography. Neurological involvement was defined as the presence of clinical findings compatible with meningitis or encephalitis after exclusion of other neurological disorders, together with at least one of the following criteria: (1) isolation of *Brucella* spp. from cerebrospinal fluid (CSF); (2) positive SAT in CSF accompanied by abnormal biochemical or cytological CSF findings; or (3) CSF abnormalities together with isolation of *Brucella* spp. from blood or bone marrow cultures. Cardiovascular involvement was defined as the presence of pericardial effusion accompanied by positive culture results or the detection of vegetations on echocardiography that could not be explained by any other cause, after exclusion of alternative etiologies (Buzgan et al., 2010; Demirdal and Sen, 2020; Arslan et al., 2024).

### 2.3 Statistical analysis

Statistical analyses were performed using SPSS version 26.0 (IBM Corp., Armonk, NY, USA). Sample size estimation was performed using G*Power version 3.1.9.7. The sample size calculation was based on the difference in ESR values between patients with and without osteoarticular involvement reported in a previous study (Bosilkovski et al., 2004). Assuming a two-sided α of 0.05, 85% power, a Cohen’s d effect size of 0.26, and a case-to-control ratio of 1:3, the minimum required sample size was estimated to be 712 patients (178 cases and 534 controls). Because the present study included 804 patients (217 cases and 587 controls), the sample size was considered adequate for the planned statistical analyses.

Continuous variables were expressed as the median and 25th–75th percentiles, whereas categorical variables were presented as frequencies and percentages. Patients with and without osteoarticular involvement were compared with respect to demographic, clinical, and laboratory characteristics. Continuous variables were compared using the Mann–Whitney U test, while categorical variables were compared using Pearson’s chi-square test or Fisher’s exact test, as appropriate.

Univariate and multivariable logistic regression analyses were performed to identify factors associated with osteoarticular involvement. The multivariable model included age, sex, symptom duration, ESR, and CRP, which were considered clinically relevant, and the Enter method was used for model construction. Multicollinearity among the independent variables was assessed using the variance inflation factor (VIF), and all variables demonstrated VIF values <5. Results were reported as odds ratios (ORs) with 95% confidence intervals (CIs). Receiver operating characteristic (ROC) curves were generated for continuous variables found to be independently associated with osteoarticular involvement, and the optimal cut-off values were determined using the Youden index.

A two-sided p value <0.05 was considered statistically significant for all analyses.

## 3 Results

### 3.1 Baseline characteristics of the study population

A total of 804 patients with brucellosis were included in the study. The median age was 45 years, and 516 (64.2%) were male. The most common presenting symptoms were arthralgia (80.1%), myalgia (76.7%), and sweating (55.0%). Additional baseline demographic and clinical characteristics are summarized in **Table 1**.

**Table 1:** Demographic and epidemiological characteristics of patients with and without osteoarticular involvement.

Variables	Total (n=804)	With osteoarticular involvement (n=217)	Without osteoarticular involvement (n=587)	P value
Age (years)	45 (34-56)	50 (34-59)	44 (33-55)	0.003
Male sex	516 (64.2)	136 (62.7)	380 (64.7)	0.588
Presence of comorbidity	155 (19.3)	50 (23)	105 (17.9)	0.100
Hypertension	88 (10.9)	34 (15.7)	54 (9.2)	0.009
Diabetes mellitus	60 (7.5)	15 (6.9)	45 (7.7)	0.718
Coronary artery disease	34 (4.2)	15 (6.9)	19 (3.2)	0.022
Chronic kidney disease	4 (0.5)	1(0.5)	3(0.5)	>0.999
Chronic lung disease	16 (2)	3 (1.4)	13 (2.2)	0.578
Malignancy	2 (0.2)	1 (0.5)	1 (0.2)	0.467
Residence in a rural area	516 (64.2)	137 (63.1)	379 (64.6)	0.707
Animal exposure	616 (76.6)	176 (81.1)	440 (75)	0.067
Consumption of unpasteurized milk and dairy products	505 (62.8)	136 (62.7)	369 (62.9)	0.961

Categorical variables are presented as n (%); numerical variables are expressed as median (25th and 75^th^ percentiles).

Osteoarticular involvement was identified in 217 (27.0%) patients, whereas genitourinary involvement, hepatic involvement, neurological involvement, and cardiovascular involvement were detected in 60 (7.5%), 17 (2.1%), 2 (0.2%), and 2 (0.2%) patients, respectively. Both patients with neurological involvement had Brucella meningitis, and both patients with cardiovascular involvement had Brucella endocarditis. Overall, 278 (34.6%) patients had hematological abnormalities. These included leukocytosis in 119 (14.8%) patients, leukopenia in 30 (3.7%), anemia in 139 (17.3%), and thrombocytopenia in 40 (5.0%).

Among patients with osteoarticular involvement, 106 (13.2%) had spondylodiscitis, 104 (12.9%) had sacroiliitis, and 28 (3.5%) had peripheral arthritis. Among patients with peripheral arthritis, the knee was the most frequently affected joint (n = 20, including one case of prosthetic knee joint infection), followed by the shoulder (n = 3), hip (n = 2), wrist (n = 2), and metacarpophalangeal joint (n = 1). Concomitant peripheral arthritis and sacroiliitis were observed in six patients, peripheral arthritis and spondylodiscitis in one patient, and sacroiliitis and spondylodiscitis in 14 patients. A total of 243 vertebral levels were affected in patients with spondylodiscitis, with 83.1% involving the lumbar spine (**Figure 1**). Among patients with spondylodiscitis, paravertebral abscesses were detected in 8 (3.7%) and psoas abscesses in 4 (1.8%).

**Figure 1:**
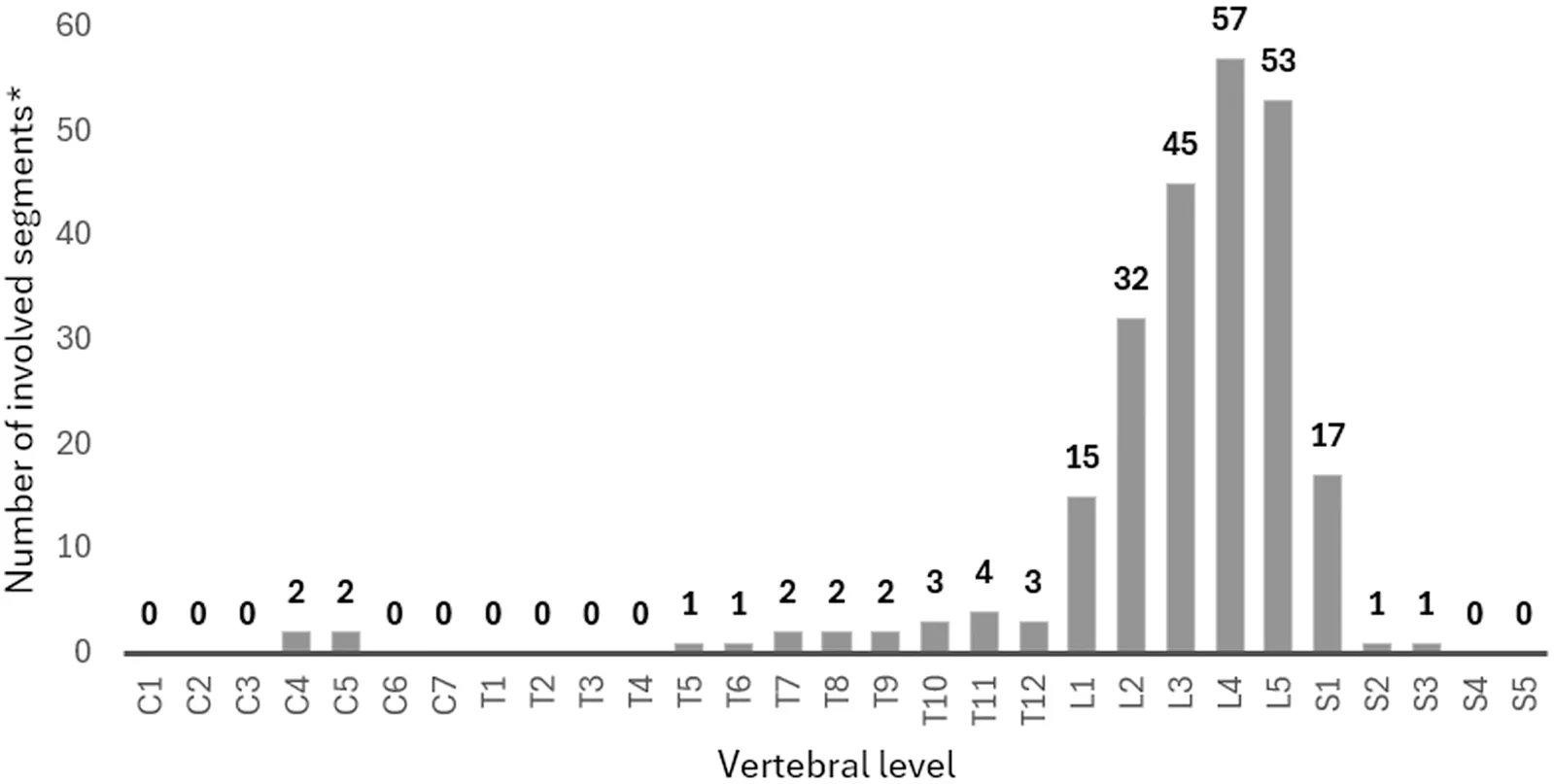
Distribution of vertebral involvement in brucellar spondylodiscitis across spinal levels (C1–S5) *Values represent the number of involved segments, not the number of patients.

### 3.2 Comparison of patients with and without osteoarticular involvement

Patients with osteoarticular involvement were significantly older and had a longer symptom duration than those without (**Tables 1**, **2**). Back pain, sacroiliac joint pain, and arthralgia were more frequent among patients with osteoarticular involvement, whereas fever was less common (**Table 2**). Both CRP and ESR were significantly higher in patients with osteoarticular involvement (**Table 3**).

**Table 2:** Clinical characteristics of patients with and without osteoarticular involvement.

Variables	Total (n=804)	With osteoarticular involvement (n=217)	Without osteoarticular involvement (n=587)	P value
Fever	318 (39.6)	68 (30.9)	251 (42.8)	0.002
Perspiration	442 (55)	111 (51.2)	331 (56.4)	0.185
Weight loss	232 (28.9)	55 (25.3)	177 (30.2)	0.182
Fatigue	372 (46.3)	98 (45.2)	274 (46.7)	0.702
Myalgia	616 (76.7)	162 (74.7)	454 (77.3)	0.424
Nausea and vomiting	161 (20)	40 (18.4)	121 (20.6)	0.493
Headache	206 (25.6)	53 (24.4)	153 (26.1)	0.636
Cough	32 (4)	2 (0.9)	30 (5.1)	0.007
Dyspnea	11 (1.4)	1 (0.5)	10 (1.7)	0.305
Abdominal pain	66 (8.2)	17 (7.8)	49 (8.3)	0.814
Scrotal pain/swelling	54 (6.7)	4 (1.8)	50 (8.5)	0.001
Skin lesion	9 (1.1)	4 (1.8)	5 (0.9)	0.236
Arthralgia	644 (80.1)	184 (84.8)	460 (78.4)	0.043
Back pain	374 (46.5)	147 (67.7)	227 (38.7)	<0.001
Sacroiliac joint pain	261 (32.5)	133 (61.3)	128 (21.8)	<0.001
Clinical stage				<0.001
Acute brucellosis	679 (84.5)	132 (60.8)	547 (93.2)	
Subacute/chronic brucellosis	125 (15.5)	85 (39.2)	40 (6.8)	
Symptom duration, weeks	3 (1-5)	6 (4-10)	2 (1-4)	<0.001
Lymphadenopathy	96 (11.9)	25 (11.5)	71 (12.1)	0.823
Hepatomegaly	80 (10)	21 (9.7)	59 (10.1)	0.875
Splenomegaly	84 (10.4)	22 (10.1)	62 (10.6)	0.862

Categorical variables are presented as n (%); numerical variables are expressed as median (25th and 75^th^ percentiles).

**Table 3:** Laboratory characteristics of patients with and without osteoarticular involvement.

Variables	Total (n=804)	With osteoarticular involvement (n=217)	Without osteoarticular involvement (n=587)	P value
White blood cell count (/mm^3^)	7205 (5960-8978)	7500 (6175-9255)	7100 (5860-8820)	0.076
Neutrophil count (/mm^3^)	3920 (3030-5105)	4200 (3265-5410)	3800 (2940-5020)	0.002
Lymphocyte count (/mm^3^)	2395 (1882.5-2950)	2390 (1880-2935)	2400 (1890-2980)	0.645
Monocyte count (/mm^3^)	560 (430-730)	600 (500-740)	540 (420-730)	0.003
Hemoglobin count (g/dL)	14.2 (12.9-15.4)	13.7 (12.6-15.2)	14.4 (13.1-15.5)	0.001
Platelet count (x10^3^/mm^3^)	255 (214-304.8)	263 (222-319)	252 (210-299)	0.021
AST (U/L)	25 (19-35)	23 (18-31)	25 (19-37)	0.002
ALT (U/L)	25 (17-40)	22 (16-33)	26 (18-45)	<0.001
Creatinine (mg/dL)	0.8 (0.7-0.9)	0.8 (0.7-0.9)	0.8 (0.7-0.9)	0.021
CRP (mg/L)	9 (2.6-31)	12 (3.2-35.7)	7.5 (2.2-30)	0.046
ESR (mm/h)	18 (8-33)	27 (12-45)	15 (8-30)	<0.001

CRP, C-reactive protein; ESR, Erythrocyte sedimentation rate; AST, Aspartate aminotransferase; ALT, Alanine aminotransferase.

Numerical variables are expressed as median (25th and 75th percentiles).

In the multivariable logistic regression analysis, only symptom duration were independently associated with osteoarticular involvement (**Table 4**). ROC curve analysis was performed for symptom duration and ESR. The AUROC for symptom duration in predicting osteoarticular involvement was 0.792 (95% CI: 0.754–0.830; p<0.001), with an optimal cut-off value of 3.5 weeks (sensitivity, 78.8%; specificity, 71.0%). The AUROC for ESR was 0.633 (95% CI: 0.589–0.677; p<0.001), with an optimal cut-off value of 22.5 mm/h (sensitivity, 59.0%; specificity, 64.1%) (**Figure 2**).

**Table 4:** Univariate and multivariable logistic regression analyses of factors associated with osteoarticular involvement.

Variables	Univariate analysis OR (95% CI)	P value	Multivariable analysis OR (95% CI)	P value
Age (years)	1.016 (1.005-1.027)	0.003	1.007 (0.995-1.018)	0.257
Male sex	0.915 (0.662-1.263)	0.588	1.296 (0.893-1.881)	0.173
Symptom duration (weeks)	1.105 (1.071-1.141)	<0.001	1.102 (1.068-1.136)	<0.001
CRP (mg/L)	1.002 (0.998-1.007)	0.326	0.998 (0.993-1.003)	0.489
ESR (mm/h)	1.022 (1.014-1.030)	<0.001	1.025 (1.016-1.034)	<0.001

CRP, C-reactive protein; ESR, Erythrocyte sedimentation rate; OR, odds ratio; CI, confidence interval.

**Figure 2:**
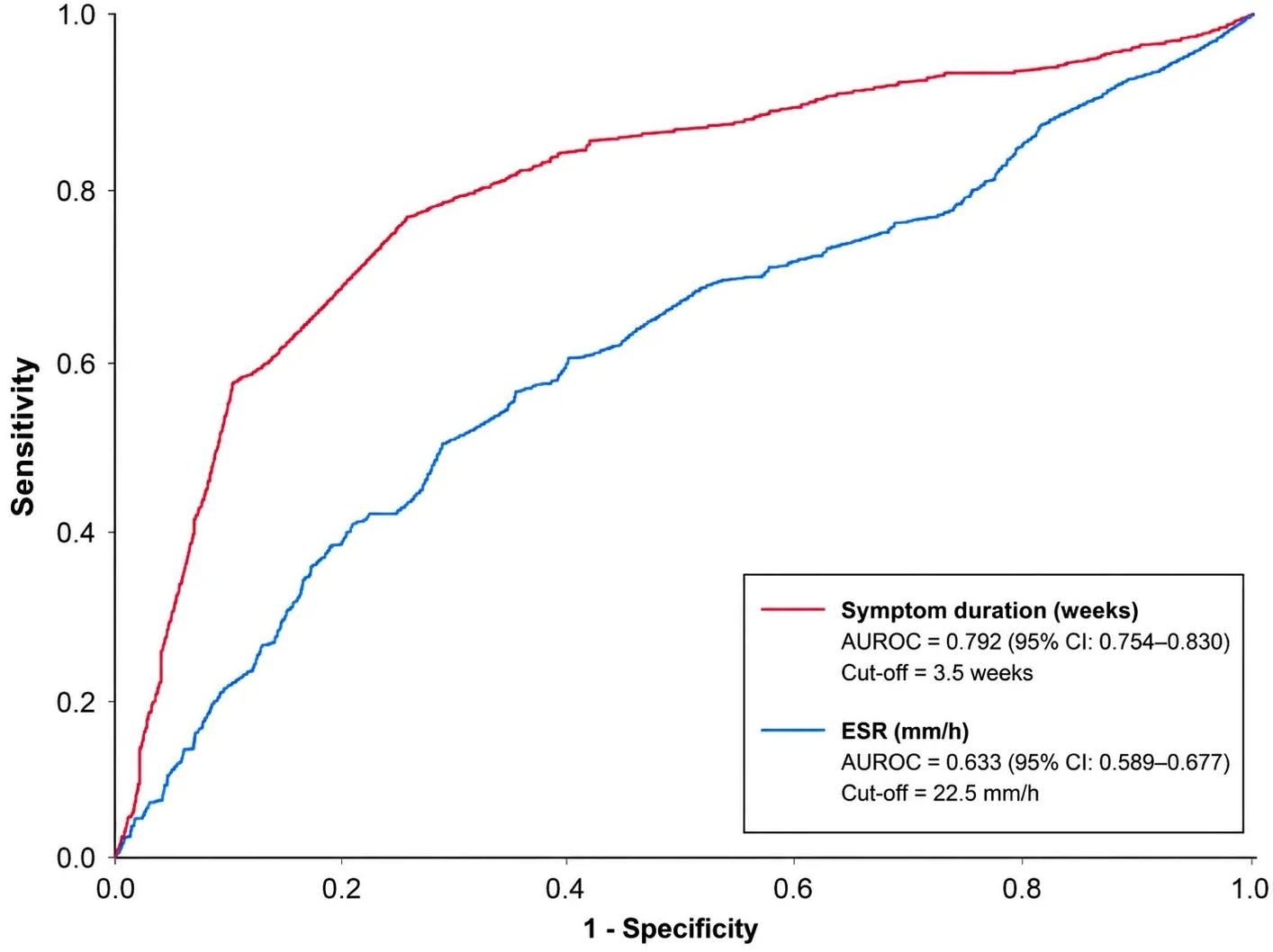
ROC curve analysis of symptom duration, and ESR, showing the optimal cut-off values associated with osteoarticular involvement. AUROC: area under receiver operating characteristic, ESR:erythrocyte sedimentation rate.

## 4 Discussion

In this study, osteoarticular involvement was identified in 27.0% of 804 patients with brucellosis. Multivariable logistic regression analysis demonstrated that symptom duration and ESR were independently associated with osteoarticular involvement. Although most previous studies have evaluated focal complications of brucellosis as a composite outcome, studies specifically investigating factors associated with osteoarticular involvement remain limited. Using data from a large multicenter cohort, the present study provides contemporary evidence on the clinical and laboratory factors associated with osteoarticular involvement and highlights readily available parameters that may facilitate the early identification of patients at increased risk. The prevalence observed in our study is consistent with the 21.4%–59.2% range reported in previous studies (Bosilkovski et al., 2004; Buzgan et al., 2010; Arslan et al., 2024; Shi et al., 2024; Dalmanoğlu et al., 2026; Gezer and Tayşi, 2026). The variability observed across studies may be attributed to differences in geographical characteristics, demographic and clinical features of the study populations, diagnostic methods used to identify osteoarticular involvement, and methodological differences in study design.

In our cohort, spondylodiscitis and sacroiliitis were the most common forms of osteoarticular involvement, whereas peripheral arthritis was less frequently observed. The knee was the most commonly affected peripheral joint, while the lumbar spine was the predominant site of vertebral involvement in patients with spondylodiscitis. These findings are consistent with previous reports on the distribution of osteoarticular brucellosis (Colmenero et al., 2008; Buzgan et al., 2010; Esmaeilnejad-Ganji and Esmaeilnejad-Ganji, 2019; Kalin Unuvar, 2019; Jin et al., 2023; Gou et al., 2024; Spernovasilis et al., 2024). The predominance of lumbar involvement in brucellar spondylodiscitis may be explained by the hematogenous dissemination of the infection and the vascular characteristics of the vertebral endplates, which predispose them to infection (Jin et al., 2023). Consistent with this anatomical distribution, back pain and sacroiliac joint pain were significantly more common in patients with osteoarticular involvement. These observations support the concept that axial skeletal pain represents an important clinical indicator of osteoarticular brucellosis.

Early recognition of osteoarticular involvement is critical in the management of brucellosis, as delayed diagnosis may lead to chronic pain, functional impairment, vertebral destruction, and neurological complications, particularly in patients with spondylodiscitis, in whom paravertebral or epidural abscesses may develop (Buzgan et al., 2010; Erdem et al., 2015; Guenifi et al., 2025; Chen et al., 2025). Osteoarticular involvement also has important therapeutic implications, often requiring prolonged antimicrobial therapy and closer clinical follow-up (Buzgan et al., 2010; Li et al., 2025). Previous studies have consistently demonstrated an association between prolonged symptom duration and focal complications of brucellosis (Dabaja-Younis et al., 2023; Shi et al., 2024). Likewise, a diagnostic delay exceeding four weeks has been reported to increase the risk of spinal involvement by approximately threefold (Pu et al., 2024). Consistent with these findings, symptom duration was independently associated with osteoarticular involvement in our study. ROC analysis identified an optimal cut-off value of 3.5 weeks, suggesting that patients with symptom durations approaching or exceeding four weeks warrant careful evaluation for possible osteoarticular involvement. *Brucella* spp. are facultative intracellular pathogens that possess multiple immune evasion mechanisms, including the ability to escape lysosomal degradation within phagocytic cells, inhibit apoptosis of infected mononuclear cells, and impair dendritic cell maturation (De Figueiredo et al., 2015). In light of these mechanisms, prolonged infection may increase bacterial burden and facilitate hematogenous dissemination to bone and joint tissues, creating a favorable environment for persistent inflammation. This biological mechanism may partially explain the observed association between prolonged symptom duration and osteoarticular involvement.

The erythrocyte sedimentation rate is a nonspecific systemic inflammatory marker that primarily reflects increases in acute-phase proteins, particularly fibrinogen, and may be influenced by a wide range of infectious and non-infectious conditions (Markanday, 2015). In osteoarticular brucellosis, persistent inflammation induced by *Brucella* spp. within bone and joint tissues is characterized by activation of various proinflammatory mediators, which contribute to bone resorption and tissue damage while sustaining the systemic acute-phase response and elevated inflammatory markers (Giambartolomei et al., 2017). Previous studies have reported higher ESR levels in patients with osteoarticular brucellosis than in those without osteoarticular involvement (Balın et al., 2018; Pu et al., 2024; Zhou et al., 2025). Consistent with these findings, ESR was independently associated with osteoarticular involvement in the present study. ROC analysis identified an optimal cut-off value of 22.5 mm/h. However, given its modest discriminatory performance, ESR should be regarded as a readily available supportive marker rather than a standalone diagnostic tool. Therefore, ESR values around 23mm/h or higher may help identify patients who warrant further evaluation for osteoarticular involvement when interpreted together with clinical findings, other laboratory results, and imaging studies.

Findings regarding the relationship between CRP and osteoarticular involvement in brucellosis have been inconsistent. Higher CRP levels have been reported in patients with osteoarticular involvement (Balın et al., 2018), whereas no association between elevated CRP and brucellar spondylitis was observed in another study (Aktug-Demir et al., 2014). Similarly, CRP was not associated with osteoarticular involvement in logistic regression analysis in our study. These findings suggest that CRP may reflect the overall inflammatory activity of brucellosis rather than the presence of osteoarticular complications specifically. The roles of age and sex also remain unclear, as previous studies have reported inconsistent findings regarding their associations with spinal or osteoarticular involvement (Aktug-Demir et al., 2014; Balın et al., 2018; Pu et al., 2024; Li et al., 2025). In our study, patients with osteoarticular involvement were older than those without such involvement; however, age did not remain independently associated after multivariable adjustment. Likewise, no association was observed with sex. These discrepancies may be attributable to differences in patient populations, patterns of occupational exposure, definitions of osteoarticular involvement, and methodological approaches across studies. Further studies are needed to clarify the influence of demographic factors on osteoarticular involvement in brucellosis.

The principal strengths of our study include its large patient population and its specific focus on factors independently associated with osteoarticular involvement. Furthermore, evaluating osteoarticular involvement separately from other focal complications enhances the clinical applicability of our findings.

Nevertheless, this study has several limitations. Owing to its retrospective design, some clinical data may have been incomplete or recorded inconsistently. In addition, imaging studies were not performed systematically in all patients, and magnetic resonance imaging was not available for every case. Consequently, some asymptomatic or mildly symptomatic cases of osteoarticular involvement may have remained undetected. Furthermore, neurological complications associated with spondylodiscitis were not systematically recorded, precluding further assessment of this association.

## 5 Conclusion

In conclusion, symptom duration and ESR were independently associated with osteoarticular involvement in patients with brucellosis. Particularly in endemic regions, patients presenting with these characteristics may warrant careful evaluation for osteoarticular complications, including appropriate imaging studies when clinically indicated. Early recognition of osteoarticular involvement may facilitate the timely initiation of appropriate treatment and contribute to the prevention of complications.

## Data Availability

The original contributions presented in the study are included in the article/supplementary material. Further inquiries can be directed to the corresponding author.
